# Didysprosium hepta­nickel

**DOI:** 10.1107/S1600536812007611

**Published:** 2012-02-24

**Authors:** Volodymyr Levytskyy, Volodymyr Babizhetskyy, Bohdan Kotur, Volodymyr Smetana

**Affiliations:** a Department of Inorganic Chemistry, Ivan Franko National University of Lviv, Kyryla & Mefodiya street 6, 79005 Lviv, Ukraine; b 344 Spedding Hall, Ames Laboratory, Ames, IA 50011-3020, USA

## Abstract

The title compound, Dy_2_Ni_7_, adopts the β-Gd_2_Co_7_-type structure type. The asymmetric unit contains two Dy sites (both site symmetry 3*m*) and five Ni sites (site symmetries .*m*, .2/*m* and -3*m*, and two 3*m*). The four different Ni coordination polyhedra are Frank–Kasper icosa­hedra formed by five Dy and seven Ni atoms, four Dy and eight Ni, three Dy and nine Ni, and six Dy and six Ni atoms, respectively. The two different Dy coordination polyhedra are either pseudo Frank–Kasper icosa­hedra formed by two Dy and 18 Ni atoms or normal Frank–Kasper icosa­hedra formed by four Dy and 12 Ni atoms.

## Related literature

For the β-Gd_2_Co_7_ structure type, see: Bertaut *et al.* (1965 ▶). For previous powder diffraction studies of the title compound, see: Lemaire *et al.* (1967 ▶); Lemaire & Paccard (1969 ▶). For related compounds, see: Buschow & van der Goot (1970 ▶). For inter­growth structures, see: Parthé *et al.* (1985 ▶); Grin (1992 ▶).

## Experimental

### 

#### Crystal data

Dy_2_Ni_7_
*M* *_r_* = 735.97
Trigonal, 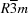

*a* = 4.9460 (9) Å
*c* = 36.191 (9) Å
*V* = 766.7 (3) Å^3^
*Z* = 6
Mo *K*α radiation
μ = 53.83 mm^−1^
*T* = 293 K
0.14 × 0.09 × 0.06 mm

#### Data collection

Stoe IPDS II diffractometer
Absorption correction: numerical (*X-RED*; Stoe & Cie, 2009 ▶) *T*_min_ = 0.067, *T*_max_ = 0.135
372 measured reflections
253 independent reflections
208 reflections with *I* > 2σ(*I*)
*R*_int_ = 0.027

#### Refinement

*R*[*F* ^2^ > 2σ(*F* ^2^)] = 0.047
*wR*(*F* ^2^) = 0.115
*S* = 1.07
253 reflections
25 parameters
Δρ_max_ = 5.07 e Å^−3^
Δρ_min_ = −2.37 e Å^−3^

### 

Data collection: *X-AREA* (Stoe & Cie, 2009 ▶); cell refinement: *X-AREA*; data reduction: *X-AREA*; program(s) used to solve structure: *SIR2011* (Burla *et al.*, 2012 ▶); program(s) used to refine structure: *SHELXL97* (Sheldrick, 2008 ▶) and *WinGX* (Farrugia, 1999 ▶); molecular graphics: *DIAMOND* (Brandenburg, 2006 ▶); software used to prepare material for publication: *publCIF* (Westrip, 2010 ▶).

## Supplementary Material

Crystal structure: contains datablock(s) I, global. DOI: 10.1107/S1600536812007611/vn2032sup1.cif

Structure factors: contains datablock(s) I. DOI: 10.1107/S1600536812007611/vn2032Isup2.hkl

Additional supplementary materials:  crystallographic information; 3D view; checkCIF report

## Figures and Tables

**Fig. 1.**
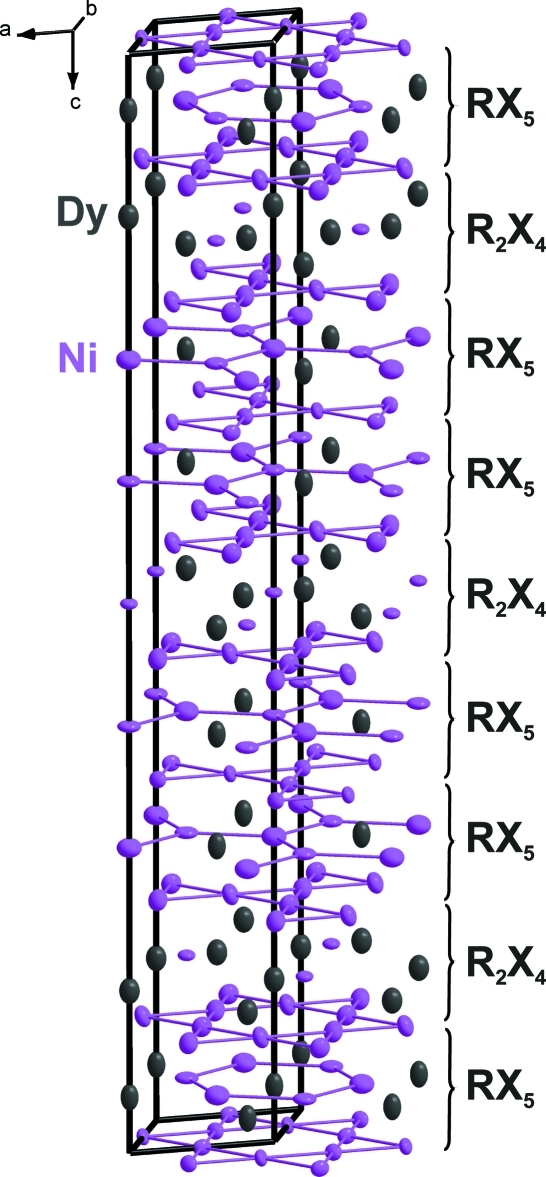
Perspective view of the crystal structure of Dy2Ni7. The unit cell and the blocks of RX5 and R2X4 are emphasized. Atoms are represented by their anisotropic displacement ellipsoids at the 99% probability level

**Fig. 2.**
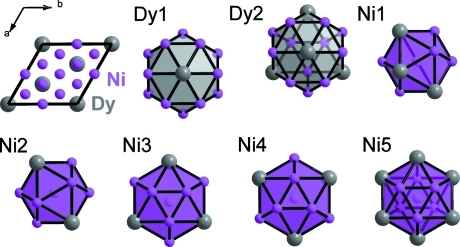
The ab projection of the unit cell and coordination polyhedra for all types of atoms in the Dy2Ni7 structure

## supplementary crystallographic information

### Comment

The existence of the Dy_2_Ni_7_ structure isotypic with the rhombohedral β-Gd_2_Co_7_ structure was reported by Lemaire *et al.* (1967) and Lemaire & Paccard (1969). The lattice parameters were determined from X-ray powder diffraction data without specifying atomic coordinates. Buschow & van der Goot (1970) prepared a series of isotypic compounds with a composition close to *R*_2_Ni_7_ (*R* = Y, La–Nd, Sm, Gd–Er) and from the X-ray powder diffraction data confirmed the lattice parameters for Dy_2_Ni_7_.

In this work we carried out a single-crystal investigation of the Dy_2_Ni_7_ inter-metallic compound. A view of the crystal structure of Dy_2_Ni_7_ is shown in Fig. 1. The structure belongs to the β-Gd_2_Co_7_ structure type (Bertaut *et al.*, 1965) and consists of stacks of *RX*_5_ blocks corresponding to the CaCu_5_-type structure and *R*_2_*X*_4_ blocks corresponding to the MgCu_2_-type structure. The presence of the same Kagome net in the structure types of CaCu_5_ and the Laves phase MgCu_2_ allows a combination of both structural motifs along the 3-fold inversion axis giving an inter-growth structure: 2*RX*_5_ + *R*_2_*X*_4_ = 2*R*_2_*X*_7_. The Kagome net serves as the common interface in the structure (Parthé *et al.*, 1985; Grin, 1992).

In Fig. 2 the *ab* projection of the unit cell and the coordination polyhedra for all atom types are shown. The coordination number for all Ni atoms is 12, but the Wyckoff site occupation is different. The coordination polyhedra are Frank–Kasper icosahedra (coordination number 12). The Ni1 atom (Wyckoff site 18*h*, site symmetry .*m*) is surrounded by 5 Dy atoms and 7 Ni atoms. The Ni2 atom (Wyckoff site 9*e*, site symmetry .2/*m*) is surrounded by 4 Dy atoms and 8 Ni atoms. The Ni3 and Ni4 atoms (both Wyckoff site 6*c*, site symmetry 3*m*) are surrounded by 3 Dy and 9 Ni atoms and by 3 Dy atoms and 9 Ni atoms respectively. The Ni5 atom (Wyckoff site 3*b*, site symmetry 3*m*) is surrounded by 6 Dy atoms and 6 Ni atoms. The coordination polyhedra for Dy1 and Dy2 atoms (both in Wyckoff site 6*c*, site symmetry 3*m*) are pseudo Frank–Kasper polyhedra (coordination number 20) and Frank–Kasper polyhedra (coordination number 16), respectively. The Dy1 atom is surrounded by 2 Dy atoms and 18 Ni atoms. The Dy2 atom is surrounded by 4 Dy atoms and 12 Ni atoms.

### Experimental

The sample was prepared from the commercially available pure elements: sublimed bulk pieces of dysprosium metal with a claimed purity of 99.99 at.% (Alfa Aesar, Johnson Matthey) and electrolytic nickel (99.99% pure) piece (Aldrich). A mixture of the powders was compacted in stainless steel dies. The pellet was arc-melted under an argon atmosphere on a water-cooled copper hearth. The alloy button (~1 g) was turned over and remelted three times to improve homogeneity. Subsequently, the sample was annealed in evacuated silica tube under an argon atmosphere for four weeks at 1070 K. Shiny metallic gray plate-like single crystals were isolated mechanically by crushing the sample.

### Refinement

The atomic positions found from the *ab initio* structure solution were in good agreement with those from the β-Gd_2_Co_7_ structure type and were used as starting point for the structure refinement. The highest Fourier difference peak of 5.07 e Å^-3^ is at (1/3 2/3 0.0305) and 0.89 Å away from the Ni4 atom. The deepest hole (-2.37 e Å^-3^) is at (1/3 2/3 0.0984) and 1.49 Å away from the Ni1 atom.

### Crystal data

**Table:**

Dy_2_Ni_7_	*D*_x_ = 9.564 Mg m^−^^3^
*M**_r_* = 735.97	Mo *K*α radiation, λ = 0.71073 Å
Trigonal, *R*3*m*	Cell parameters from 1802 reflections
Hall symbol: -R 3 2"	θ = 1.6–27.2°
*a* = 4.9460 (9) Å	µ = 53.83 mm^−^^1^
*c* = 36.191 (9) Å	*T* = 293 K
*V* = 766.7 (3) Å^3^	Plate-like, grey
*Z* = 6	0.14 × 0.09 × 0.06 mm
*F*(000) = 1968	

### Data collection

**Table:**

Stoe IPDS II diffractometer	253 independent reflections
Radiation source: fine-focus sealed tube	208 reflections with *I* > 2σ(*I*)
Graphite monochromator	*R*_int_ = 0.027
ω scans	θ_max_ = 26.7°, θ_min_ = 1.7°
Absorption correction: numerical (*X-RED*; Stoe & Cie, 2009)	*h* = −6→3
*T*_min_ = 0.067, *T*_max_ = 0.135	*k* = 0→6
372 measured reflections	*l* = 0→45

### Refinement

**Table:**

Refinement on *F*^2^	0 restraints
Least-squares matrix: full	Primary atom site location: structure-invariant direct methods
*R*[*F*^2^ > 2σ(*F*^2^)] = 0.047	Secondary atom site location: difference Fourier map
*wR*(*F*^2^) = 0.115	*w* = 1/[σ^2^(*F*_o_^2^) + (0.075*P*)^2^] where *P* = (*F*_o_^2^ + 2*F*_c_^2^)/3
*S* = 1.07	(Δ/σ)_max_ < 0.001
253 reflections	Δρ_max_ = 5.07 e Å^−^^3^
25 parameters	Δρ_min_ = −2.37 e Å^−^^3^

### Special details

**Table:**

Geometry. All e.s.d.'s (except the e.s.d. in the dihedral angle between two l.s. planes) are estimated using the full covariance matrix. The cell e.s.d.'s are taken into account individually in the estimation of e.s.d.'s in distances, angles and torsion angles; correlations between e.s.d.'s in cell parameters are only used when they are defined by crystal symmetry. An approximate (isotropic) treatment of cell e.s.d.'s is used for estimating e.s.d.'s involving l.s. planes.
Refinement. Refinement of *F*^2^ against ALL reflections. The weighted *R*-factor *wR* and goodness of fit *S* are based on *F*^2^, conventional *R*-factors *R* are based on *F*, with *F* set to zero for negative *F*^2^. The threshold expression of *F*^2^ > σ(*F*^2^) is used only for calculating *R*-factors(gt) *etc*. and is not relevant to the choice of reflections for refinement. *R*-factors based on *F*^2^ are statistically about twice as large as those based on *F*, and *R*-factors based on ALL data will be even larger.

### Fractional atomic coordinates and isotropic or equivalent isotropic displacement parameters (Å^2^)

**Table:**

	*x*	*y*	*z*	*U*_iso_*/*U*_eq_	
Dy1	0.0000	0.0000	0.05083 (5)	0.0143 (5)	
Dy2	0.0000	0.0000	0.14764 (5)	0.0152 (5)	
Ni1	0.5005 (3)	0.4995 (3)	0.10972 (7)	0.0124 (7)	
Ni2	0.5000	0.0000	0.0000	0.0096 (8)	
Ni3	0.0000	0.0000	0.27796 (13)	0.0158 (11)	
Ni4	0.0000	0.0000	0.38831 (12)	0.0133 (10)	
Ni5	0.0000	0.0000	0.5000	0.0097 (13)	

### Atomic displacement parameters (Å^2^)

**Table:**

	*U*^11^	*U*^22^	*U*^33^	*U*^12^	*U*^13^	*U*^23^
Dy1	0.0103 (6)	0.0103 (6)	0.0222 (9)	0.0052 (3)	0.000	0.000
Dy2	0.0129 (7)	0.0129 (7)	0.0199 (9)	0.0065 (3)	0.000	0.000
Ni1	0.0128 (10)	0.0128 (10)	0.0137 (11)	0.0080 (11)	0.0007 (5)	−0.0007 (5)
Ni2	0.0102 (12)	0.0080 (18)	0.0099 (14)	0.0040 (9)	0.0001 (6)	0.0003 (12)
Ni3	0.0167 (16)	0.0167 (16)	0.014 (2)	0.0083 (8)	0.000	0.000
Ni4	0.0158 (15)	0.0158 (15)	0.0083 (19)	0.0079 (7)	0.000	0.000
Ni5	0.0102 (19)	0.0102 (19)	0.009 (3)	0.0051 (10)	0.000	0.000

### Geometric parameters (Å, º)

**Table:**

Dy1—Ni4^i^	2.8595 (6)	Ni2—Ni2^xix^	2.4730 (4)
Dy1—Ni4^ii^	2.8595 (6)	Ni2—Dy1^xx^	3.0821 (11)
Dy1—Ni4^iii^	2.8595 (6)	Ni2—Dy1^xxi^	3.0821 (11)
Dy1—Ni3^iv^	2.8603 (6)	Ni2—Dy1^xxii^	3.0821 (11)
Dy1—Ni3^v^	2.8603 (6)	Ni3—Ni1^iv^	2.428 (4)
Dy1—Ni3^vi^	2.8603 (6)	Ni3—Ni1^xvi^	2.428 (4)
Dy1—Ni2^vii^	3.0821 (11)	Ni3—Ni1^xv^	2.428 (4)
Dy1—Ni2^viii^	3.0821 (11)	Ni3—Ni2^xxiii^	2.461 (4)
Dy1—Ni2^ix^	3.0821 (11)	Ni3—Ni2^xxiv^	2.461 (4)
Dy1—Ni2^x^	3.0821 (11)	Ni3—Ni2^xxv^	2.461 (4)
Dy1—Ni2	3.0821 (11)	Ni3—Ni4^xxvi^	2.8556 (5)
Dy1—Ni2^xi^	3.0821 (11)	Ni3—Ni4^xxvii^	2.8556 (5)
Dy2—Ni1^xii^	2.8283 (14)	Ni3—Ni4^xxviii^	2.8556 (5)
Dy2—Ni1^xi^	2.8283 (14)	Ni3—Dy1^iv^	2.8603 (6)
Dy2—Ni1^vii^	2.8283 (14)	Ni3—Dy1^v^	2.8603 (6)
Dy2—Ni1	2.8283 (14)	Ni3—Dy1^vi^	2.8603 (6)
Dy2—Ni1^xiii^	2.8283 (14)	Ni4—Ni1^xxix^	2.445 (4)
Dy2—Ni1^xiv^	2.8283 (14)	Ni4—Ni1^xxx^	2.445 (4)
Dy2—Ni5^i^	2.9374 (7)	Ni4—Ni1^xxxi^	2.445 (4)
Dy2—Ni5^ii^	2.9374 (7)	Ni4—Ni2^xxiii^	2.449 (3)
Dy2—Ni5^iii^	2.9374 (7)	Ni4—Ni2^xxv^	2.449 (3)
Dy2—Ni1^iv^	3.096 (3)	Ni4—Ni2^xxiv^	2.449 (3)
Dy2—Ni1^xv^	3.096 (3)	Ni4—Ni3^xxvi^	2.8556 (5)
Dy2—Ni1^xvi^	3.096 (3)	Ni4—Ni3^xxvii^	2.8556 (5)
Ni1—Ni3^iv^	2.428 (4)	Ni4—Ni3^xxviii^	2.8556 (5)
Ni1—Ni4^i^	2.445 (4)	Ni4—Dy1^xxix^	2.8595 (6)
Ni1—Ni1^x^	2.465 (5)	Ni4—Dy1^xxxii^	2.8595 (6)
Ni1—Ni1^xiv^	2.465 (5)	Ni4—Dy1^xxiv^	2.8595 (6)
Ni1—Ni1^xiii^	2.481 (5)	Ni5—Ni1^xxvi^	2.510 (3)
Ni1—Ni1^xvii^	2.481 (5)	Ni5—Ni1^xxix^	2.510 (3)
Ni1—Ni5^i^	2.510 (3)	Ni5—Ni1^xxxiii^	2.510 (3)
Ni1—Dy2^xviii^	2.8283 (14)	Ni5—Ni1^xxxi^	2.510 (3)
Ni1—Dy2^iv^	3.096 (3)	Ni5—Ni1^xxxiv^	2.510 (3)
Ni1—Dy1^xviii^	3.265 (2)	Ni5—Ni1^xxx^	2.510 (3)
Ni2—Ni4^iv^	2.449 (3)	Ni5—Dy2^xxvi^	2.9374 (7)
Ni2—Ni4^iii^	2.449 (3)	Ni5—Dy2^xxix^	2.9374 (7)
Ni2—Ni3^iv^	2.461 (4)	Ni5—Dy2^xxvii^	2.9374 (7)
Ni2—Ni3^iii^	2.461 (4)	Ni5—Dy2^xxxii^	2.9374 (7)
Ni2—Ni2^xiv^	2.4730 (5)	Ni5—Dy2^xxviii^	2.9374 (7)
Ni2—Ni2^viii^	2.4730 (5)	Ni5—Dy2^xxiv^	2.9374 (7)
Ni2—Ni2^x^	2.4730 (5)		
			
Ni4^i^—Dy1—Ni4^ii^	119.729 (16)	Ni2^viii^—Ni2—Dy1^xx^	113.652 (8)
Ni4^i^—Dy1—Ni4^iii^	119.727 (16)	Ni2^x^—Ni2—Dy1^xx^	113.652 (8)
Ni4^ii^—Dy1—Ni4^iii^	119.727 (16)	Ni2^xix^—Ni2—Dy1^xx^	66.348 (8)
Ni4^i^—Dy1—Ni3^iv^	59.900 (4)	Ni4^iv^—Ni2—Dy1^xxi^	61.00 (3)
Ni4^ii^—Dy1—Ni3^iv^	173.70 (14)	Ni4^iii^—Ni2—Dy1^xxi^	119.00 (3)
Ni4^iii^—Dy1—Ni3^iv^	59.901 (4)	Ni3^iv^—Ni2—Dy1^xxi^	119.08 (3)
Ni4^i^—Dy1—Ni3^v^	173.70 (14)	Ni3^iii^—Ni2—Dy1^xxi^	60.92 (3)
Ni4^ii^—Dy1—Ni3^v^	59.900 (4)	Ni2^xiv^—Ni2—Dy1^xxi^	113.652 (8)
Ni4^iii^—Dy1—Ni3^v^	59.901 (4)	Ni2^viii^—Ni2—Dy1^xxi^	66.348 (8)
Ni3^iv^—Dy1—Ni3^v^	119.67 (2)	Ni2^x^—Ni2—Dy1^xxi^	66.348 (8)
Ni4^i^—Dy1—Ni3^vi^	59.901 (4)	Ni2^xix^—Ni2—Dy1^xxi^	113.652 (8)
Ni4^ii^—Dy1—Ni3^vi^	59.901 (4)	Dy1^xx^—Ni2—Dy1^xxi^	180.00 (5)
Ni4^iii^—Dy1—Ni3^vi^	173.70 (14)	Ni4^iv^—Ni2—Dy1	119.00 (3)
Ni3^iv^—Dy1—Ni3^vi^	119.67 (2)	Ni4^iii^—Ni2—Dy1	61.00 (3)
Ni3^v^—Dy1—Ni3^vi^	119.67 (2)	Ni3^iv^—Ni2—Dy1	60.92 (3)
Ni4^i^—Dy1—Ni2^vii^	136.49 (8)	Ni3^iii^—Ni2—Dy1	119.08 (3)
Ni4^ii^—Dy1—Ni2^vii^	48.50 (7)	Ni2^xiv^—Ni2—Dy1	113.652 (8)
Ni4^iii^—Dy1—Ni2^vii^	91.79 (5)	Ni2^viii^—Ni2—Dy1	66.348 (8)
Ni3^iv^—Dy1—Ni2^vii^	136.72 (9)	Ni2^x^—Ni2—Dy1	66.348 (8)
Ni3^v^—Dy1—Ni2^vii^	48.75 (8)	Ni2^xix^—Ni2—Dy1	113.652 (8)
Ni3^vi^—Dy1—Ni2^vii^	91.97 (6)	Dy1^xx^—Ni2—Dy1	106.71 (5)
Ni4^i^—Dy1—Ni2^viii^	136.49 (8)	Dy1^xxi^—Ni2—Dy1	73.29 (5)
Ni4^ii^—Dy1—Ni2^viii^	91.79 (5)	Ni4^iv^—Ni2—Dy1^xxii^	61.00 (3)
Ni4^iii^—Dy1—Ni2^viii^	48.50 (7)	Ni4^iii^—Ni2—Dy1^xxii^	119.00 (3)
Ni3^iv^—Dy1—Ni2^viii^	91.97 (6)	Ni3^iv^—Ni2—Dy1^xxii^	119.08 (3)
Ni3^v^—Dy1—Ni2^viii^	48.75 (8)	Ni3^iii^—Ni2—Dy1^xxii^	60.92 (3)
Ni3^vi^—Dy1—Ni2^viii^	136.72 (9)	Ni2^xiv^—Ni2—Dy1^xxii^	66.348 (8)
Ni2^vii^—Dy1—Ni2^viii^	47.304 (17)	Ni2^viii^—Ni2—Dy1^xxii^	113.652 (8)
Ni4^i^—Dy1—Ni2^ix^	91.79 (5)	Ni2^x^—Ni2—Dy1^xxii^	113.652 (8)
Ni4^ii^—Dy1—Ni2^ix^	48.50 (7)	Ni2^xix^—Ni2—Dy1^xxii^	66.348 (8)
Ni4^iii^—Dy1—Ni2^ix^	136.49 (8)	Dy1^xx^—Ni2—Dy1^xxii^	73.29 (5)
Ni3^iv^—Dy1—Ni2^ix^	136.72 (9)	Dy1^xxi^—Ni2—Dy1^xxii^	106.71 (5)
Ni3^v^—Dy1—Ni2^ix^	91.97 (6)	Dy1—Ni2—Dy1^xxii^	180.00 (5)
Ni3^vi^—Dy1—Ni2^ix^	48.75 (8)	Ni1^iv^—Ni3—Ni1^xvi^	61.02 (15)
Ni2^vii^—Dy1—Ni2^ix^	47.304 (17)	Ni1^iv^—Ni3—Ni1^xv^	61.02 (15)
Ni2^viii^—Dy1—Ni2^ix^	88.03 (4)	Ni1^xvi^—Ni3—Ni1^xv^	61.02 (15)
Ni4^i^—Dy1—Ni2^x^	48.50 (7)	Ni1^iv^—Ni3—Ni2^xxiii^	108.64 (7)
Ni4^ii^—Dy1—Ni2^x^	136.49 (8)	Ni1^xvi^—Ni3—Ni2^xxiii^	146.09 (3)
Ni4^iii^—Dy1—Ni2^x^	91.79 (5)	Ni1^xv^—Ni3—Ni2^xxiii^	146.09 (3)
Ni3^iv^—Dy1—Ni2^x^	48.75 (8)	Ni1^iv^—Ni3—Ni2^xxiv^	146.09 (3)
Ni3^v^—Dy1—Ni2^x^	136.72 (9)	Ni1^xvi^—Ni3—Ni2^xxiv^	146.09 (3)
Ni3^vi^—Dy1—Ni2^x^	91.97 (6)	Ni1^xv^—Ni3—Ni2^xxiv^	108.64 (7)
Ni2^vii^—Dy1—Ni2^x^	106.71 (5)	Ni2^xxiii^—Ni3—Ni2^xxiv^	60.33 (11)
Ni2^viii^—Dy1—Ni2^x^	88.03 (4)	Ni1^iv^—Ni3—Ni2^xxv^	146.09 (3)
Ni2^ix^—Dy1—Ni2^x^	88.03 (4)	Ni1^xvi^—Ni3—Ni2^xxv^	108.64 (7)
Ni4^i^—Dy1—Ni2	91.79 (5)	Ni1^xv^—Ni3—Ni2^xxv^	146.09 (3)
Ni4^ii^—Dy1—Ni2	136.49 (8)	Ni2^xxiii^—Ni3—Ni2^xxv^	60.33 (11)
Ni4^iii^—Dy1—Ni2	48.50 (7)	Ni2^xxiv^—Ni3—Ni2^xxv^	60.33 (11)
Ni3^iv^—Dy1—Ni2	48.75 (8)	Ni1^iv^—Ni3—Ni4^xxvi^	107.29 (14)
Ni3^v^—Dy1—Ni2	91.97 (6)	Ni1^xvi^—Ni3—Ni4^xxvi^	107.29 (14)
Ni3^vi^—Dy1—Ni2	136.72 (9)	Ni1^xv^—Ni3—Ni4^xxvi^	54.40 (11)
Ni2^vii^—Dy1—Ni2	88.03 (4)	Ni2^xxiii^—Ni3—Ni4^xxvi^	106.62 (14)
Ni2^viii^—Dy1—Ni2	47.304 (17)	Ni2^xxiv^—Ni3—Ni4^xxvi^	54.24 (9)
Ni2^ix^—Dy1—Ni2	106.71 (5)	Ni2^xxv^—Ni3—Ni4^xxvi^	106.62 (14)
Ni2^x^—Dy1—Ni2	47.304 (17)	Ni1^iv^—Ni3—Ni4^xxvii^	107.29 (14)
Ni4^i^—Dy1—Ni2^xi^	48.50 (7)	Ni1^xvi^—Ni3—Ni4^xxvii^	54.40 (11)
Ni4^ii^—Dy1—Ni2^xi^	91.79 (5)	Ni1^xv^—Ni3—Ni4^xxvii^	107.29 (14)
Ni4^iii^—Dy1—Ni2^xi^	136.49 (8)	Ni2^xxiii^—Ni3—Ni4^xxvii^	106.62 (14)
Ni3^iv^—Dy1—Ni2^xi^	91.97 (6)	Ni2^xxiv^—Ni3—Ni4^xxvii^	106.62 (14)
Ni3^v^—Dy1—Ni2^xi^	136.72 (9)	Ni2^xxv^—Ni3—Ni4^xxvii^	54.24 (9)
Ni3^vi^—Dy1—Ni2^xi^	48.75 (8)	Ni4^xxvi^—Ni3—Ni4^xxvii^	119.998 (3)
Ni2^vii^—Dy1—Ni2^xi^	88.03 (4)	Ni1^iv^—Ni3—Ni4^xxviii^	54.40 (11)
Ni2^viii^—Dy1—Ni2^xi^	106.71 (5)	Ni1^xvi^—Ni3—Ni4^xxviii^	107.29 (14)
Ni2^ix^—Dy1—Ni2^xi^	47.304 (17)	Ni1^xv^—Ni3—Ni4^xxviii^	107.29 (14)
Ni2^x^—Dy1—Ni2^xi^	47.304 (17)	Ni2^xxiii^—Ni3—Ni4^xxviii^	54.24 (9)
Ni2—Dy1—Ni2^xi^	88.03 (4)	Ni2^xxiv^—Ni3—Ni4^xxviii^	106.62 (14)
Ni1^xii^—Dy2—Ni1^xi^	52.03 (11)	Ni2^xxv^—Ni3—Ni4^xxviii^	106.62 (14)
Ni1^xii^—Dy2—Ni1^vii^	51.67 (11)	Ni4^xxvi^—Ni3—Ni4^xxviii^	119.997 (3)
Ni1^xi^—Dy2—Ni1^vii^	98.44 (6)	Ni4^xxvii^—Ni3—Ni4^xxviii^	119.997 (3)
Ni1^xii^—Dy2—Ni1	121.94 (10)	Ni1^iv^—Ni3—Dy1^iv^	75.76 (7)
Ni1^xi^—Dy2—Ni1	98.44 (6)	Ni1^xvi^—Ni3—Dy1^iv^	129.19 (18)
Ni1^vii^—Dy2—Ni1	98.44 (6)	Ni1^xv^—Ni3—Dy1^iv^	75.76 (7)
Ni1^xii^—Dy2—Ni1^xiii^	98.44 (6)	Ni2^xxiii^—Ni3—Dy1^iv^	70.34 (6)
Ni1^xi^—Dy2—Ni1^xiii^	51.67 (11)	Ni2^xxiv^—Ni3—Dy1^iv^	70.34 (6)
Ni1^vii^—Dy2—Ni1^xiii^	121.94 (10)	Ni2^xxv^—Ni3—Dy1^iv^	122.17 (17)
Ni1—Dy2—Ni1^xiii^	52.03 (11)	Ni4^xxvi^—Ni3—Dy1^iv^	60.035 (7)
Ni1^xii^—Dy2—Ni1^xiv^	98.44 (6)	Ni4^xxvii^—Ni3—Dy1^iv^	176.4 (2)
Ni1^xi^—Dy2—Ni1^xiv^	121.94 (10)	Ni4^xxviii^—Ni3—Dy1^iv^	60.036 (7)
Ni1^vii^—Dy2—Ni1^xiv^	52.03 (11)	Ni1^iv^—Ni3—Dy1^v^	75.76 (7)
Ni1—Dy2—Ni1^xiv^	51.67 (11)	Ni1^xvi^—Ni3—Dy1^v^	75.76 (7)
Ni1^xiii^—Dy2—Ni1^xiv^	98.44 (6)	Ni1^xv^—Ni3—Dy1^v^	129.19 (18)
Ni1^xii^—Dy2—Ni5^i^	148.28 (6)	Ni2^xxiii^—Ni3—Dy1^v^	70.34 (6)
Ni1^xi^—Dy2—Ni5^i^	96.44 (6)	Ni2^xxiv^—Ni3—Dy1^v^	122.17 (17)
Ni1^vii^—Dy2—Ni5^i^	148.28 (6)	Ni2^xxv^—Ni3—Dy1^v^	70.34 (6)
Ni1—Dy2—Ni5^i^	51.56 (5)	Ni4^xxvi^—Ni3—Dy1^v^	176.4 (2)
Ni1^xiii^—Dy2—Ni5^i^	51.56 (5)	Ni4^xxvii^—Ni3—Dy1^v^	60.035 (7)
Ni1^xiv^—Dy2—Ni5^i^	96.44 (6)	Ni4^xxviii^—Ni3—Dy1^v^	60.036 (7)
Ni1^xii^—Dy2—Ni5^ii^	51.56 (5)	Dy1^iv^—Ni3—Dy1^v^	119.67 (2)
Ni1^xi^—Dy2—Ni5^ii^	51.56 (5)	Ni1^iv^—Ni3—Dy1^vi^	129.19 (18)
Ni1^vii^—Dy2—Ni5^ii^	96.44 (6)	Ni1^xvi^—Ni3—Dy1^vi^	75.76 (7)
Ni1—Dy2—Ni5^ii^	148.28 (6)	Ni1^xv^—Ni3—Dy1^vi^	75.76 (7)
Ni1^xiii^—Dy2—Ni5^ii^	96.44 (6)	Ni2^xxiii^—Ni3—Dy1^vi^	122.17 (17)
Ni1^xiv^—Dy2—Ni5^ii^	148.28 (6)	Ni2^xxiv^—Ni3—Dy1^vi^	70.34 (6)
Ni5^i^—Dy2—Ni5^ii^	114.68 (3)	Ni2^xxv^—Ni3—Dy1^vi^	70.34 (6)
Ni1^xii^—Dy2—Ni5^iii^	96.44 (6)	Ni4^xxvi^—Ni3—Dy1^vi^	60.036 (7)
Ni1^xi^—Dy2—Ni5^iii^	148.28 (6)	Ni4^xxvii^—Ni3—Dy1^vi^	60.036 (7)
Ni1^vii^—Dy2—Ni5^iii^	51.56 (5)	Ni4^xxviii^—Ni3—Dy1^vi^	176.4 (2)
Ni1—Dy2—Ni5^iii^	96.44 (6)	Dy1^iv^—Ni3—Dy1^vi^	119.67 (2)
Ni1^xiii^—Dy2—Ni5^iii^	148.28 (6)	Dy1^v^—Ni3—Dy1^vi^	119.67 (2)
Ni1^xiv^—Dy2—Ni5^iii^	51.56 (5)	Ni1^xxix^—Ni4—Ni1^xxx^	60.97 (14)
Ni5^i^—Dy2—Ni5^iii^	114.68 (3)	Ni1^xxix^—Ni4—Ni1^xxxi^	60.97 (14)
Ni5^ii^—Dy2—Ni5^iii^	114.68 (3)	Ni1^xxx^—Ni4—Ni1^xxxi^	60.97 (14)
Ni1^xii^—Dy2—Ni1^iv^	115.48 (5)	Ni1^xxix^—Ni4—Ni2^xxiii^	108.47 (6)
Ni1^xi^—Dy2—Ni1^iv^	141.21 (5)	Ni1^xxx^—Ni4—Ni2^xxiii^	146.02 (3)
Ni1^vii^—Dy2—Ni1^iv^	94.77 (8)	Ni1^xxxi^—Ni4—Ni2^xxiii^	146.02 (3)
Ni1—Dy2—Ni1^iv^	115.48 (5)	Ni1^xxix^—Ni4—Ni2^xxv^	146.02 (3)
Ni1^xiii^—Dy2—Ni1^iv^	141.21 (5)	Ni1^xxx^—Ni4—Ni2^xxv^	146.02 (3)
Ni1^xiv^—Dy2—Ni1^iv^	94.77 (8)	Ni1^xxxi^—Ni4—Ni2^xxv^	108.47 (7)
Ni5^i^—Dy2—Ni1^iv^	90.88 (5)	Ni2^xxiii^—Ni4—Ni2^xxv^	60.66 (9)
Ni5^ii^—Dy2—Ni1^iv^	90.88 (5)	Ni1^xxix^—Ni4—Ni2^xxiv^	146.02 (3)
Ni5^iii^—Dy2—Ni1^iv^	49.08 (5)	Ni1^xxx^—Ni4—Ni2^xxiv^	108.47 (7)
Ni1^xii^—Dy2—Ni1^xv^	141.21 (5)	Ni1^xxxi^—Ni4—Ni2^xxiv^	146.02 (3)
Ni1^xi^—Dy2—Ni1^xv^	115.48 (5)	Ni2^xxiii^—Ni4—Ni2^xxiv^	60.66 (9)
Ni1^vii^—Dy2—Ni1^xv^	141.21 (5)	Ni2^xxv^—Ni4—Ni2^xxiv^	60.66 (9)
Ni1—Dy2—Ni1^xv^	94.77 (8)	Ni1^xxix^—Ni4—Ni3^xxvi^	106.79 (14)
Ni1^xiii^—Dy2—Ni1^xv^	94.77 (8)	Ni1^xxx^—Ni4—Ni3^xxvi^	53.85 (12)
Ni1^xiv^—Dy2—Ni1^xv^	115.48 (5)	Ni1^xxxi^—Ni4—Ni3^xxvi^	106.79 (14)
Ni5^i^—Dy2—Ni1^xv^	49.08 (5)	Ni2^xxiii^—Ni4—Ni3^xxvi^	107.19 (13)
Ni5^ii^—Dy2—Ni1^xv^	90.88 (5)	Ni2^xxv^—Ni4—Ni3^xxvi^	107.20 (13)
Ni5^iii^—Dy2—Ni1^xv^	90.88 (5)	Ni2^xxiv^—Ni4—Ni3^xxvi^	54.62 (10)
Ni1^iv^—Dy2—Ni1^xv^	46.92 (9)	Ni1^xxix^—Ni4—Ni3^xxvii^	106.79 (14)
Ni1^xii^—Dy2—Ni1^xvi^	94.77 (8)	Ni1^xxx^—Ni4—Ni3^xxvii^	106.79 (14)
Ni1^xi^—Dy2—Ni1^xvi^	94.77 (8)	Ni1^xxxi^—Ni4—Ni3^xxvii^	53.85 (12)
Ni1^vii^—Dy2—Ni1^xvi^	115.48 (5)	Ni2^xxiii^—Ni4—Ni3^xxvii^	107.19 (13)
Ni1—Dy2—Ni1^xvi^	141.21 (5)	Ni2^xxv^—Ni4—Ni3^xxvii^	54.62 (10)
Ni1^xiii^—Dy2—Ni1^xvi^	115.48 (5)	Ni2^xxiv^—Ni4—Ni3^xxvii^	107.20 (13)
Ni1^xiv^—Dy2—Ni1^xvi^	141.21 (5)	Ni3^xxvi^—Ni4—Ni3^xxvii^	119.998 (3)
Ni5^i^—Dy2—Ni1^xvi^	90.88 (5)	Ni1^xxix^—Ni4—Ni3^xxviii^	53.85 (12)
Ni5^ii^—Dy2—Ni1^xvi^	49.08 (5)	Ni1^xxx^—Ni4—Ni3^xxviii^	106.79 (14)
Ni5^iii^—Dy2—Ni1^xvi^	90.88 (5)	Ni1^xxxi^—Ni4—Ni3^xxviii^	106.79 (14)
Ni1^iv^—Dy2—Ni1^xvi^	46.92 (9)	Ni2^xxiii^—Ni4—Ni3^xxviii^	54.62 (10)
Ni1^xv^—Dy2—Ni1^xvi^	46.92 (9)	Ni2^xxv^—Ni4—Ni3^xxviii^	107.20 (13)
Ni3^iv^—Ni1—Ni4^i^	71.75 (10)	Ni2^xxiv^—Ni4—Ni3^xxviii^	107.20 (13)
Ni3^iv^—Ni1—Ni1^x^	59.49 (7)	Ni3^xxvi^—Ni4—Ni3^xxviii^	119.997 (3)
Ni4^i^—Ni1—Ni1^x^	120.49 (7)	Ni3^xxvii^—Ni4—Ni3^xxviii^	119.997 (3)
Ni3^iv^—Ni1—Ni1^xiv^	59.49 (7)	Ni1^xxix^—Ni4—Dy1^xxix^	75.52 (6)
Ni4^i^—Ni1—Ni1^xiv^	120.49 (7)	Ni1^xxx^—Ni4—Dy1^xxix^	128.87 (16)
Ni1^x^—Ni1—Ni1^xiv^	60.0	Ni1^xxxi^—Ni4—Dy1^xxix^	75.52 (6)
Ni3^iv^—Ni1—Ni1^xiii^	120.51 (7)	Ni2^xxiii^—Ni4—Dy1^xxix^	70.51 (6)
Ni4^i^—Ni1—Ni1^xiii^	59.51 (7)	Ni2^xxv^—Ni4—Dy1^xxix^	70.51 (6)
Ni1^x^—Ni1—Ni1^xiii^	180.00 (13)	Ni2^xxiv^—Ni4—Dy1^xxix^	122.66 (14)
Ni1^xiv^—Ni1—Ni1^xiii^	120.000 (1)	Ni3^xxvi^—Ni4—Dy1^xxix^	177.3 (2)
Ni3^iv^—Ni1—Ni1^xvii^	120.51 (7)	Ni3^xxvii^—Ni4—Dy1^xxix^	60.063 (7)
Ni4^i^—Ni1—Ni1^xvii^	59.51 (7)	Ni3^xxviii^—Ni4—Dy1^xxix^	60.064 (7)
Ni1^x^—Ni1—Ni1^xvii^	120.000 (1)	Ni1^xxix^—Ni4—Dy1^xxxii^	75.52 (6)
Ni1^xiv^—Ni1—Ni1^xvii^	180.00 (13)	Ni1^xxx^—Ni4—Dy1^xxxii^	75.52 (6)
Ni1^xiii^—Ni1—Ni1^xvii^	60.000 (1)	Ni1^xxxi^—Ni4—Dy1^xxxii^	128.87 (16)
Ni3^iv^—Ni1—Ni5^i^	178.91 (14)	Ni2^xxiii^—Ni4—Dy1^xxxii^	70.51 (6)
Ni4^i^—Ni1—Ni5^i^	109.34 (12)	Ni2^xxv^—Ni4—Dy1^xxxii^	122.66 (14)
Ni1^x^—Ni1—Ni5^i^	119.62 (5)	Ni2^xxiv^—Ni4—Dy1^xxxii^	70.51 (6)
Ni1^xiv^—Ni1—Ni5^i^	119.62 (5)	Ni3^xxvi^—Ni4—Dy1^xxxii^	60.063 (7)
Ni1^xiii^—Ni1—Ni5^i^	60.38 (5)	Ni3^xxvii^—Ni4—Dy1^xxxii^	177.3 (2)
Ni1^xvii^—Ni1—Ni5^i^	60.38 (5)	Ni3^xxviii^—Ni4—Dy1^xxxii^	60.064 (7)
Ni3^iv^—Ni1—Dy2^xviii^	113.21 (6)	Dy1^xxix^—Ni4—Dy1^xxxii^	119.728 (17)
Ni4^i^—Ni1—Dy2^xviii^	113.10 (7)	Ni1^xxix^—Ni4—Dy1^xxiv^	128.86 (16)
Ni1^x^—Ni1—Dy2^xviii^	64.16 (6)	Ni1^xxx^—Ni4—Dy1^xxiv^	75.52 (6)
Ni1^xiv^—Ni1—Dy2^xviii^	116.01 (6)	Ni1^xxxi^—Ni4—Dy1^xxiv^	75.52 (6)
Ni1^xiii^—Ni1—Dy2^xviii^	115.84 (5)	Ni2^xxiii^—Ni4—Dy1^xxiv^	122.66 (14)
Ni1^xvii^—Ni1—Dy2^xviii^	63.99 (6)	Ni2^xxv^—Ni4—Dy1^xxiv^	70.50 (6)
Ni5^i^—Ni1—Dy2^xviii^	66.46 (5)	Ni2^xxiv^—Ni4—Dy1^xxiv^	70.50 (6)
Ni3^iv^—Ni1—Dy2	113.21 (6)	Ni3^xxvi^—Ni4—Dy1^xxiv^	60.064 (7)
Ni4^i^—Ni1—Dy2	113.10 (7)	Ni3^xxvii^—Ni4—Dy1^xxiv^	60.064 (7)
Ni1^x^—Ni1—Dy2	116.01 (6)	Ni3^xxviii^—Ni4—Dy1^xxiv^	177.3 (2)
Ni1^xiv^—Ni1—Dy2	64.16 (6)	Dy1^xxix^—Ni4—Dy1^xxiv^	119.727 (17)
Ni1^xiii^—Ni1—Dy2	63.99 (6)	Dy1^xxxii^—Ni4—Dy1^xxiv^	119.727 (17)
Ni1^xvii^—Ni1—Dy2	115.84 (5)	Ni1^xxvi^—Ni5—Ni1^xxix^	180.00 (6)
Ni5^i^—Ni1—Dy2	66.46 (5)	Ni1^xxvi^—Ni5—Ni1^xxxiii^	59.24 (10)
Dy2^xviii^—Ni1—Dy2	121.94 (10)	Ni1^xxix^—Ni5—Ni1^xxxiii^	120.76 (10)
Ni3^iv^—Ni1—Dy2^iv^	116.74 (12)	Ni1^xxvi^—Ni5—Ni1^xxxi^	120.76 (10)
Ni4^i^—Ni1—Dy2^iv^	171.51 (13)	Ni1^xxix^—Ni5—Ni1^xxxi^	59.24 (10)
Ni1^x^—Ni1—Dy2^iv^	66.54 (4)	Ni1^xxxiii^—Ni5—Ni1^xxxi^	180.000 (1)
Ni1^xiv^—Ni1—Dy2^iv^	66.54 (4)	Ni1^xxvi^—Ni5—Ni1^xxxiv^	59.24 (10)
Ni1^xiii^—Ni1—Dy2^iv^	113.46 (4)	Ni1^xxix^—Ni5—Ni1^xxxiv^	120.76 (10)
Ni1^xvii^—Ni1—Dy2^iv^	113.46 (4)	Ni1^xxxiii^—Ni5—Ni1^xxxiv^	59.24 (10)
Ni5^i^—Ni1—Dy2^iv^	62.17 (6)	Ni1^xxxi^—Ni5—Ni1^xxxiv^	120.76 (11)
Dy2^xviii^—Ni1—Dy2^iv^	64.52 (5)	Ni1^xxvi^—Ni5—Ni1^xxx^	120.76 (10)
Dy2—Ni1—Dy2^iv^	64.52 (5)	Ni1^xxix^—Ni5—Ni1^xxx^	59.24 (10)
Ni3^iv^—Ni1—Dy1	58.12 (5)	Ni1^xxxiii^—Ni5—Ni1^xxx^	120.76 (11)
Ni4^i^—Ni1—Dy1	58.00 (5)	Ni1^xxxi^—Ni5—Ni1^xxx^	59.24 (10)
Ni1^x^—Ni1—Dy1	112.33 (5)	Ni1^xxxiv^—Ni5—Ni1^xxx^	180.000 (1)
Ni1^xiv^—Ni1—Dy1	67.82 (5)	Ni1^xxvi^—Ni5—Dy2^xxvi^	61.98 (3)
Ni1^xiii^—Ni1—Dy1	67.67 (5)	Ni1^xxix^—Ni5—Dy2^xxvi^	118.02 (3)
Ni1^xvii^—Ni1—Dy1	112.18 (5)	Ni1^xxxiii^—Ni5—Dy2^xxvi^	61.98 (3)
Ni5^i^—Ni1—Dy1	122.36 (6)	Ni1^xxxi^—Ni5—Dy2^xxvi^	118.02 (3)
Dy2^xviii^—Ni1—Dy1	168.27 (8)	Ni1^xxxiv^—Ni5—Dy2^xxvi^	111.25 (7)
Dy2—Ni1—Dy1	69.79 (4)	Ni1^xxx^—Ni5—Dy2^xxvi^	68.75 (7)
Dy2^iv^—Ni1—Dy1	125.48 (5)	Ni1^xxvi^—Ni5—Dy2^xxix^	118.02 (3)
Ni3^iv^—Ni1—Dy1^xviii^	58.12 (5)	Ni1^xxix^—Ni5—Dy2^xxix^	61.98 (3)
Ni4^i^—Ni1—Dy1^xviii^	58.00 (5)	Ni1^xxxiii^—Ni5—Dy2^xxix^	118.03 (3)
Ni1^x^—Ni1—Dy1^xviii^	67.82 (5)	Ni1^xxxi^—Ni5—Dy2^xxix^	61.97 (3)
Ni1^xiv^—Ni1—Dy1^xviii^	112.33 (5)	Ni1^xxxiv^—Ni5—Dy2^xxix^	68.75 (7)
Ni1^xiii^—Ni1—Dy1^xviii^	112.18 (5)	Ni1^xxx^—Ni5—Dy2^xxix^	111.25 (7)
Ni1^xvii^—Ni1—Dy1^xviii^	67.67 (5)	Dy2^xxvi^—Ni5—Dy2^xxix^	180.0
Ni5^i^—Ni1—Dy1^xviii^	122.36 (6)	Ni1^xxvi^—Ni5—Dy2^xxvii^	61.98 (3)
Dy2^xviii^—Ni1—Dy1^xviii^	69.79 (4)	Ni1^xxix^—Ni5—Dy2^xxvii^	118.02 (3)
Dy2—Ni1—Dy1^xviii^	168.27 (8)	Ni1^xxxiii^—Ni5—Dy2^xxvii^	111.25 (7)
Dy2^iv^—Ni1—Dy1^xviii^	125.48 (5)	Ni1^xxxi^—Ni5—Dy2^xxvii^	68.75 (7)
Dy1—Ni1—Dy1^xviii^	98.49 (8)	Ni1^xxxiv^—Ni5—Dy2^xxvii^	61.98 (3)
Ni4^iv^—Ni2—Ni4^iii^	180.00 (11)	Ni1^xxx^—Ni5—Dy2^xxvii^	118.02 (3)
Ni4^iv^—Ni2—Ni3^iv^	108.86 (8)	Dy2^xxvi^—Ni5—Dy2^xxvii^	114.68 (3)
Ni4^iii^—Ni2—Ni3^iv^	71.14 (8)	Dy2^xxix^—Ni5—Dy2^xxvii^	65.32 (3)
Ni4^iv^—Ni2—Ni3^iii^	71.14 (8)	Ni1^xxvi^—Ni5—Dy2^xxxii^	118.02 (3)
Ni4^iii^—Ni2—Ni3^iii^	108.86 (8)	Ni1^xxix^—Ni5—Dy2^xxxii^	61.98 (3)
Ni3^iv^—Ni2—Ni3^iii^	180.00 (13)	Ni1^xxxiii^—Ni5—Dy2^xxxii^	68.75 (7)
Ni4^iv^—Ni2—Ni2^xiv^	59.67 (5)	Ni1^xxxi^—Ni5—Dy2^xxxii^	111.25 (7)
Ni4^iii^—Ni2—Ni2^xiv^	120.33 (5)	Ni1^xxxiv^—Ni5—Dy2^xxxii^	118.03 (3)
Ni3^iv^—Ni2—Ni2^xiv^	59.83 (5)	Ni1^xxx^—Ni5—Dy2^xxxii^	61.97 (3)
Ni3^iii^—Ni2—Ni2^xiv^	120.17 (5)	Dy2^xxvi^—Ni5—Dy2^xxxii^	65.32 (3)
Ni4^iv^—Ni2—Ni2^viii^	120.33 (5)	Dy2^xxix^—Ni5—Dy2^xxxii^	114.68 (3)
Ni4^iii^—Ni2—Ni2^viii^	59.67 (5)	Dy2^xxvii^—Ni5—Dy2^xxxii^	180.00 (7)
Ni3^iv^—Ni2—Ni2^viii^	120.17 (5)	Ni1^xxvi^—Ni5—Dy2^xxviii^	111.24 (7)
Ni3^iii^—Ni2—Ni2^viii^	59.83 (5)	Ni1^xxix^—Ni5—Dy2^xxviii^	68.76 (7)
Ni2^xiv^—Ni2—Ni2^viii^	180.0	Ni1^xxxiii^—Ni5—Dy2^xxviii^	61.97 (3)
Ni4^iv^—Ni2—Ni2^x^	59.67 (5)	Ni1^xxxi^—Ni5—Dy2^xxviii^	118.03 (3)
Ni4^iii^—Ni2—Ni2^x^	120.33 (5)	Ni1^xxxiv^—Ni5—Dy2^xxviii^	61.97 (3)
Ni3^iv^—Ni2—Ni2^x^	59.83 (5)	Ni1^xxx^—Ni5—Dy2^xxviii^	118.03 (3)
Ni3^iii^—Ni2—Ni2^x^	120.17 (5)	Dy2^xxvi^—Ni5—Dy2^xxviii^	114.68 (3)
Ni2^xiv^—Ni2—Ni2^x^	60.0	Dy2^xxix^—Ni5—Dy2^xxviii^	65.32 (3)
Ni2^viii^—Ni2—Ni2^x^	120.0	Dy2^xxvii^—Ni5—Dy2^xxviii^	114.68 (3)
Ni4^iv^—Ni2—Ni2^xix^	120.33 (5)	Dy2^xxxii^—Ni5—Dy2^xxviii^	65.32 (3)
Ni4^iii^—Ni2—Ni2^xix^	59.67 (5)	Ni1^xxvi^—Ni5—Dy2^xxiv^	68.76 (7)
Ni3^iv^—Ni2—Ni2^xix^	120.17 (5)	Ni1^xxix^—Ni5—Dy2^xxiv^	111.24 (7)
Ni3^iii^—Ni2—Ni2^xix^	59.83 (5)	Ni1^xxxiii^—Ni5—Dy2^xxiv^	118.03 (3)
Ni2^xiv^—Ni2—Ni2^xix^	120.0	Ni1^xxxi^—Ni5—Dy2^xxiv^	61.97 (3)
Ni2^viii^—Ni2—Ni2^xix^	60.0	Ni1^xxxiv^—Ni5—Dy2^xxiv^	118.03 (3)
Ni2^x^—Ni2—Ni2^xix^	180.0	Ni1^xxx^—Ni5—Dy2^xxiv^	61.97 (3)
Ni4^iv^—Ni2—Dy1^xx^	119.00 (3)	Dy2^xxvi^—Ni5—Dy2^xxiv^	65.32 (3)
Ni4^iii^—Ni2—Dy1^xx^	61.00 (3)	Dy2^xxix^—Ni5—Dy2^xxiv^	114.68 (3)
Ni3^iv^—Ni2—Dy1^xx^	60.92 (3)	Dy2^xxvii^—Ni5—Dy2^xxiv^	65.32 (3)
Ni3^iii^—Ni2—Dy1^xx^	119.08 (3)	Dy2^xxxii^—Ni5—Dy2^xxiv^	114.68 (3)
Ni2^xiv^—Ni2—Dy1^xx^	66.348 (8)	Dy2^xxviii^—Ni5—Dy2^xxiv^	180.00 (7)

Symmetry codes: (i) *x*+1/3, *y*+2/3, *z*−1/3; (ii) *x*−2/3, *y*−1/3, *z*−1/3; (iii) *x*+1/3, *y*−1/3, *z*−1/3; (iv) −*x*+2/3, −*y*+1/3, −*z*+1/3; (v) −*x*−1/3, −*y*−2/3, −*z*+1/3; (vi) −*x*−1/3, −*y*+1/3, −*z*+1/3; (vii) −*x*+*y*, −*x*, *z*; (viii) −*y*, *x*−*y*−1, *z*; (ix) *x*−1, *y*, *z*; (x) −*x*+*y*+1, −*x*+1, *z*; (xi) −*y*, *x*−*y*, *z*; (xii) *x*−1, *y*−1, *z*; (xiii) −*x*+*y*, −*x*+1, *z*; (xiv) −*y*+1, *x*−*y*, *z*; (xv) *y*−1/3, −*x*+*y*+1/3, −*z*+1/3; (xvi) *x*−*y*−1/3, *x*−2/3, −*z*+1/3; (xvii) −*y*+1, *x*−*y*+1, *z*; (xviii) *x*+1, *y*+1, *z*; (xix) −*x*+*y*+1, −*x*, *z*; (xx) *x*+1, *y*, *z*; (xxi) −*x*, −*y*, −*z*; (xxii) −*x*+1, −*y*, −*z*; (xxiii) −*x*+*y*+2/3, −*x*+1/3, *z*+1/3; (xxiv) *x*−1/3, *y*+1/3, *z*+1/3; (xxv) −*y*−1/3, *x*−*y*−2/3, *z*+1/3; (xxvi) −*x*+1/3, −*y*+2/3, −*z*+2/3; (xxvii) −*x*−2/3, −*y*−1/3, −*z*+2/3; (xxviii) −*x*+1/3, −*y*−1/3, −*z*+2/3; (xxix) *x*−1/3, *y*−2/3, *z*+1/3; (xxx) −*y*+2/3, *x*−*y*+1/3, *z*+1/3; (xxxi) −*x*+*y*−1/3, −*x*+1/3, *z*+1/3; (xxxii) *x*+2/3, *y*+1/3, *z*+1/3; (xxxiii) *x*−*y*+1/3, *x*−1/3, −*z*+2/3; (xxxiv) *y*−2/3, −*x*+*y*−1/3, −*z*+2/3.
